# Evaluation of the impact of AI-based chatbot on orthodontic patient education: a preliminary randomised controlled trial

**DOI:** 10.1007/s00784-025-06356-8

**Published:** 2025-04-30

**Authors:** Simona Santonocito, Marco Cicciù, Vincenzo Ronsivalle

**Affiliations:** 1 Department of Biomedical and Dental Sciences and Morphological and Functional Imaging, University of Messina, Messina, Italy; 2 Department of General Surgery and Surgical-Medical Specialties, School of dentistry, University of Catania, Catania, Italy

**Keywords:** Artificial Intelligence, LLMs, AI-based chatbot, Orthodontics, Dental monitoring, Oral hygiene, Orthodontic treatment

## Abstract

**Objectives:**

To evaluate the impact of AI-based chatbots on orthodontic patient education in terms of compliance with oral hygiene procedures and level of knowledge and understanding of the treatment recommendations received. Furthermore, to assess the patient’s satisfaction with the information received.

**Materials and methods:**

100 orthodontic patients were enrolled and randomly allocated to receive information leaflets (control group *n* = 50) or access to an AI-based chatbot created on the guidelines of the leading scientific societies in the field (*n* = 50). The plaque index (PI) and modified gingival index (MGI) were assessed at baseline (T0) and after 5 weeks of treatment (T1). A questionnaire with a Likert scale was used to evaluate patients’ knowledge and satisfaction. Statistical investigations were conducted to perform intra- and inter-group evaluations and to compare the effects of orthodontic therapies on the independent variables analysed. The questionnaire’ s reliability was assessed using Cronbach’s α.

**Results:**

At T1, a statistically significant increase in MGI and PI was observed in both analyzed groups (*P* < 0,001). However, the increase in MGI in the chatbot group was statistically lower than in the control group (*P* < 0.001). The increase in MGI was significantly higher in the chatbot-fixed orthodontic treatment subgroup than in the chatbot-aligners subgroup (*P* < 0,001).

**Conclusions:**

The use of AI-based chatbots, whose reliability of the information provided can be validated, positively influences orthodontic oral hygiene in orthodontic patients. Further studies with greater follow-up should be conducted to understand the real impact of AI-based chatbot on patient education and satisfaction.

## Introduction

In orthodontics, patient collaboration regarding adherence to oral hygiene procedures and treatment recommendations is crucial for achieving therapeutic goals and optimising treatment time [1, 2]. Beyond sociodemographic factors, patient compliance is significantly influenced by the effective communication of information regarding oral hygiene and treatment protocols [3]. Traditionally, this information has been provided by orthodontists; however, with the rise of digital tools, it is now increasingly accessible through online platforms. More recently, Artificial Intelligence (AI)-based chatbots have emerged as a new source of patient education [4–6]. These AI-based chatbots, developed using Large Language Model (LLM), are neural network-based language models trained on vast text datasets from the internet to process prompts and generate coherent, human-like conversational responses [7]. Today, AI chatbots have gained widespread popularity, offering convenient, accessible sources of information for self-care [8]. However, while these platforms provide quick answers, the reliability and accuracy of their information—especially concerning human health—remains a concern. The precision of health-related information is critical, and in this context, AI chatbots may not always meet the standards necessary for patient well-being [4, 8, 9]. Recent studies have indicated that although precision and accuracy on certain topics is high, current available chatbots (ChatGPT, Google Gemini, Bing of Microsoft) cannot be recognised as medical information devices, so they should not be used indiscriminately by individuals with medical inexperience [7, 10–12]. In another comparative study, the responses of three chatbot platforms (ChatGPT- 4, Microsoft Copilot and Google Gemini) on twenty orthodontic-related queries were evaluated in terms of completeness and accuracy, indicating that although these chatbots generally handle basic orthodontic queries well, they show significant differences in their responses to complex scenarios. Furthermore, ChatGPT- 4 and Microsoft Copilot outperform Google Gemini in accurately addressing scenario-based queries, highlighting the importance of continued improvements in chatbot technology [13]. The lack of clear regulations regarding chatbots in the healthcare sector, especially in areas such as information quality, ethical considerations, and responsibility, significantly hinders efforts to assess their impact on patient knowledge, treatment adherence, and satisfaction [14]. Without clear guidelines, the potential for misinformation is heightened, especially in critical healthcare settings, where inaccurate advice can lead to patient harm, improper treatment decisions, or delayed care. This regulatory gap raises concerns about the potential for unintended consequences in critical healthcare settings, further complicating the integration of AI chatbots into patient care [4]. Previous research has explored the use of AI-driven remote monitoring technology to enhance orthodontic patients’ oral hygiene through customized active notification [15]. However, the direct impact of AI-based chatbot systems to investigate the interactive and informational support that AI could provide for orthodontic knowledge and patient compliance remains poorly understood. This gap underscores the need for further research into how these systems can be improved and regulated to ensure their safe and effective use in patient education.

In light of the above, the primary objective of this study is to evaluate the impact of an AI-based chatbot on orthodontic patient education in terms of compliance with oral hygiene procedures. The secondary objective is to assess the level of knowledge and understanding of the therapeutic recommendations received and the degree of patient satisfaction with the information received.

## Materials and methods

**Fig. 1:**
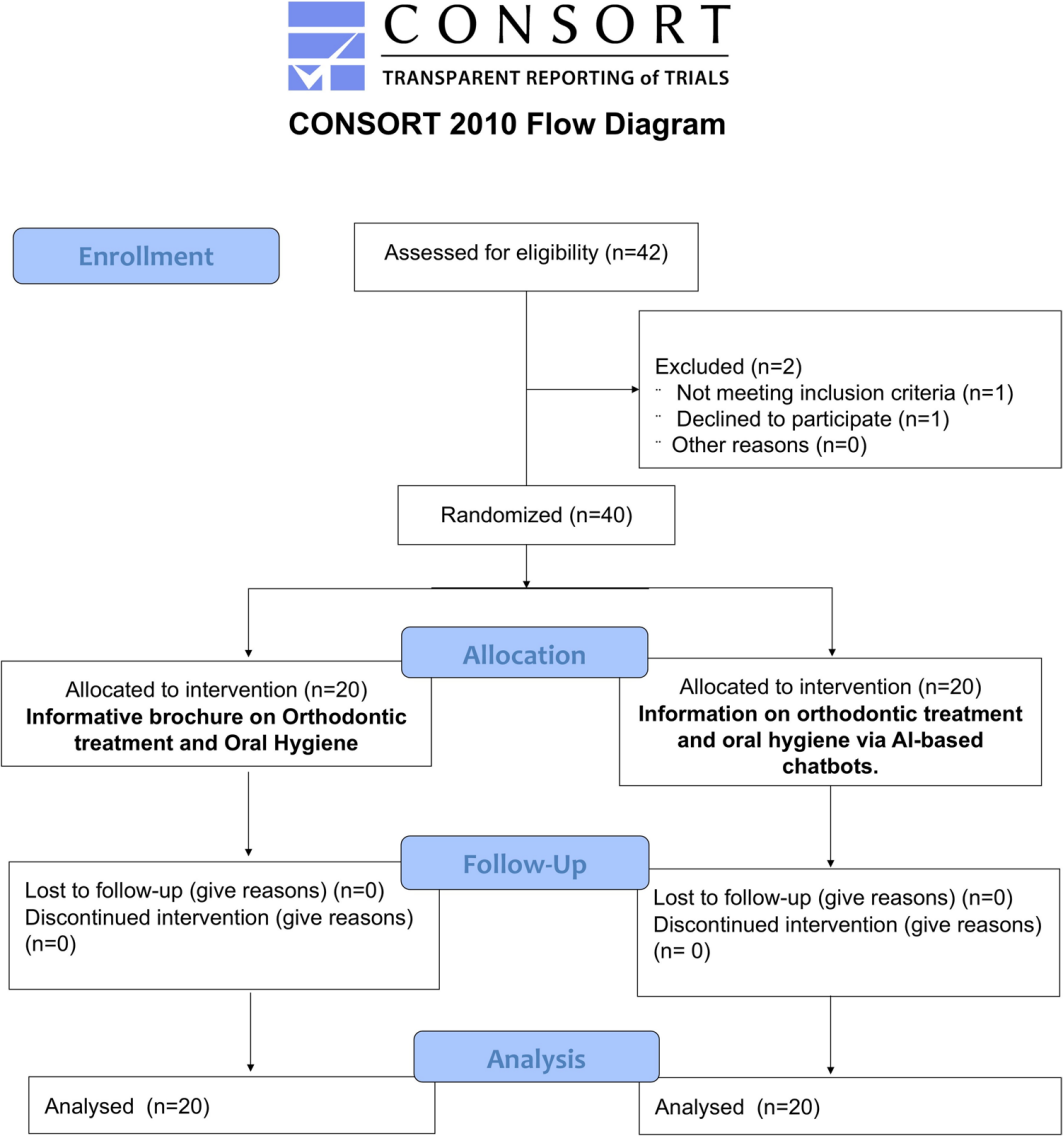
Flow-chat of the study. The figure was created with Power Point (Microsoft 365 package, Microsoft Corporation, Washington, USA)

The study was designed as a randomised controlled clinical trial (RCT) according to Consort 2010 guidelines (Fig. 1) and it was conducted in accordance with the 2016 revision of Declaration of Helsinki. It was registered at the Local Ethics Committee with a number protocol 60/2024/CL-PAR. Written informed consent was obtained from all participants prior to the use of their data.

### Study design

A sample of 100 patients were enrolled at private studios located in the province of Catania, between June 2023 and August 2024.

The eligibility criteria were:

- age ≥ 18 years;
- undergoing orthodontic treatment with labially placed fixed brackets or aligner therapy;
- daily access to a smartphone.

The exclusion criteria were:

- age < 18 years;
- patients awaiting orthognathic surgery;
- undergoing extractive orthodontic treatment;
- presence of untreated or active periodontal disease;
- currently undergoing orthodontic therapy;
- patients with cognitive deficits.

All patients were informed about the nature of the study, through informed consent. At the end of the study, the chatbot was also made available to the control group.

Patients were randomized into 2 groups (50 for each arm) of interventions, according to random allocation generated in a 1:1 ratio, using a computer-based randomization sequence (Randomization.com):

- Chatbot group: patients used an AI-based chatbot via a QR code to ask questions and receive guidance on oral hygiene and orthodontic treatment.
- Control group: received standard educational material by means of information leaflets, compiled by the leading scientific societies in orthodontics (*Sido – Italian Society of Orthodontics, American Association of Orthodontists, AAO*).

The randomization process was conducted by an operator who was not involved in any part of the study, ensuring that allocation concealment was maintained throughout the process. Use of automated software for randomisation reduces the risk of bias through strictly controlling the assignment process and prevents any influence or manipulation by the researchers. This automated approach ensured that the allocation to each intervention group remained random, transparent, and free from any researcher bias or external interference.

To assess the impact of orthodontic therapy on the two groups analyzed, four subgroups were identified:

- Leaflet-fixed therapy (LF) with 27 patients,
- Leaflet-aligner (LA) with 23 patients,
- Chatbot -fixed therapy (CF) with 24 patients,
- Chatbot-aligner (CA) with 26 patients.

### Data collection

Patients’ knowledge of and compliance with treatment recommendations and oral hygiene procedures were assessed by means of a clinical examination, performed at baseline (T0) and 5 weeks after the start of orthodontic treatment (T1), and a questionnaire.

### Clinical examination

**Fig. 2:**
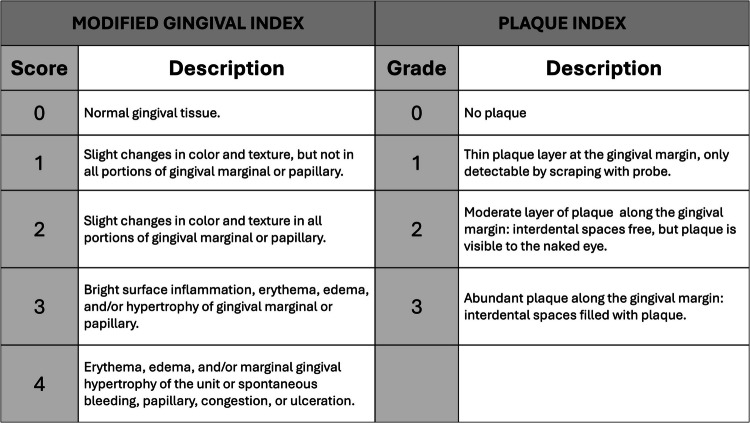
Description of Modified Gingival Index (GMI) and Plaque index (PI). The figure was created with Power Point (Microsoft 365 package, Microsoft Corporation, Washington, USA)
- Plaque Index (PI) of Silness & Löe 1964 scores range from 0 to 3, evaluating plaque thickness along the gingival margin. A score of 0 indicates no plaque, while 3 reflects plaque presence along the gingival margin and interdental papillae involvement [1, 17] (Fig. 2).

Each patient underwent a clinical examination by an experienced operator who was not involved in the study. The examination assessed compliance with oral hygiene and treatment guidelines by measuring the following parameters at baseline (T0) and 5 weeks after the start of treatment (T1), using a UCN 15 mm periodontal probe (Hu-Friedy, Italy S.R.L.- Milan):

- Modified Gingival Index (MGI) scores range from 0 to 4, where 0 indicates healthy gums and 4 represents severe gingival inflammation with spontaneous bleeding [15, 16] (Fig. 2);

Each tooth was assessed at six sites (mesiobuccal, buccal, distobuccal, distolingual, lingual, and mesiolingual). The average score of all evaluated teeth was calculated for each patient. Two operators, not involved in the study, performed the measurements after calibration. Calibration was considered successful if repeated measurements had a 95% agreement.

### Questionnaire

**Fig. 3:**
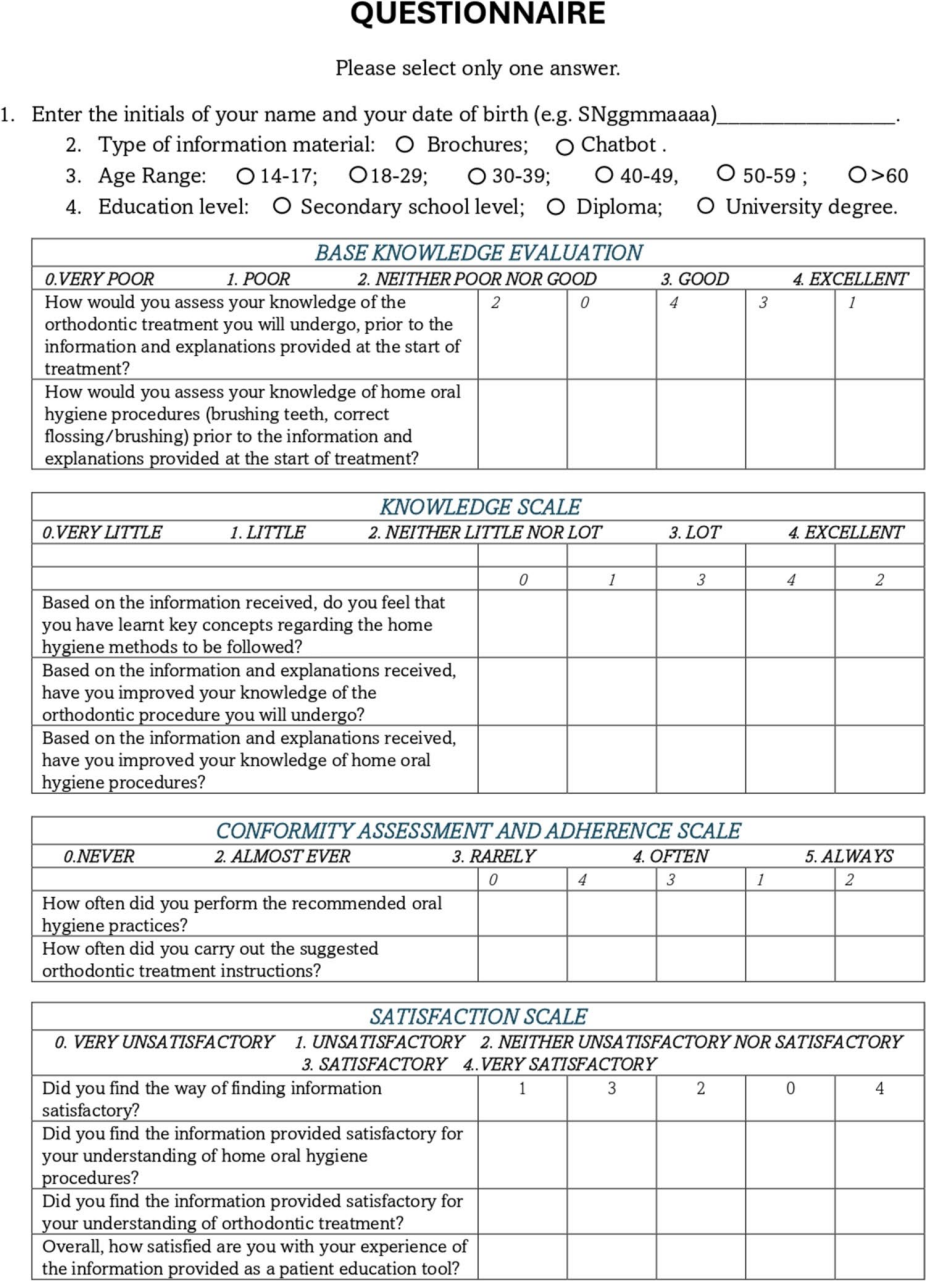
Fac-simile of the questionnaire. The figure was created with Power Point (Microsoft 365 package, Microsoft Corporation, Washington, USA)

At T1, each patient completed an online questionnaire accessed via a QR code. The questionnaire was created using Microsoft Forms and linked to Excel datasets (Microsoft 365, Microsoft Corporation, Washington, USA). It consisted of four sections: Knowledge Evaluation (KE_S), Understanding Scale (US_S), Compliance Assessment and Adherence Scale (CAAS_S), and Satisfaction Scale (SS_S) (Fig. 3). The questionnaire used a 5-point Likert scale [18], with responses scored from 0 to 4, where 0 indicates strongly negative, 1 negative, 2 neutral, 3 positive, and 4 strongly positive (Fig. 3). To minimise bias and automatic responses, the order of polarities in some questions has been reversed [18]. Each section score represented the participant’s attitude toward that specific aspect (KE_S, US_S, CAAS_S, and SS_S) and was calculated by summing the individual question scores. The total score for each participant, representing their overall attitude toward the intervention (control group or chatbot group), was derived by summing the scores across all sections (Q_TOT).

### Creation of chatbots

**Fig. 4:**
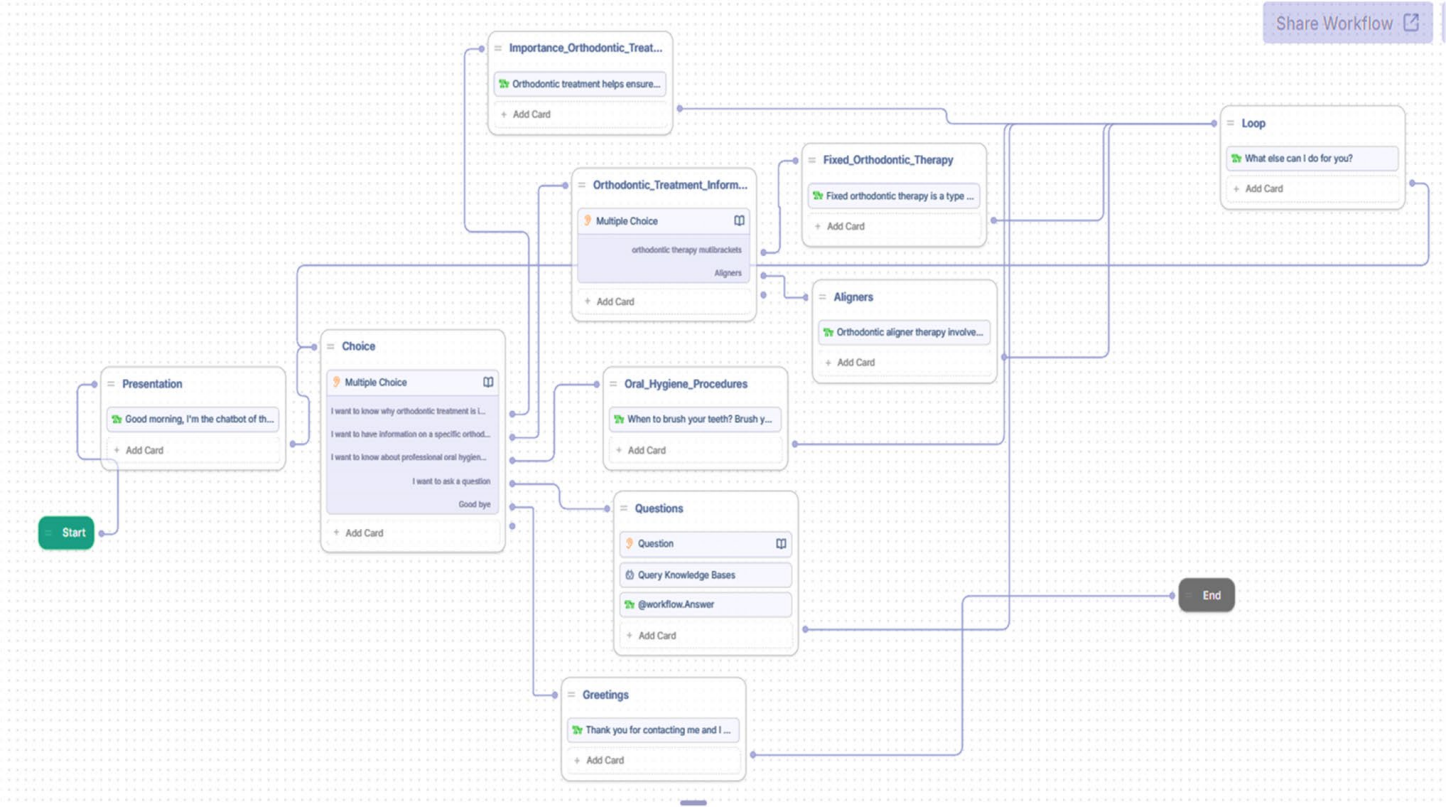
Creation of the chatbot algorithm. The figure was created with Power Point (Microsoft 365 package, Microsoft Corporation, Washington, USA)

**Fig. 5:**
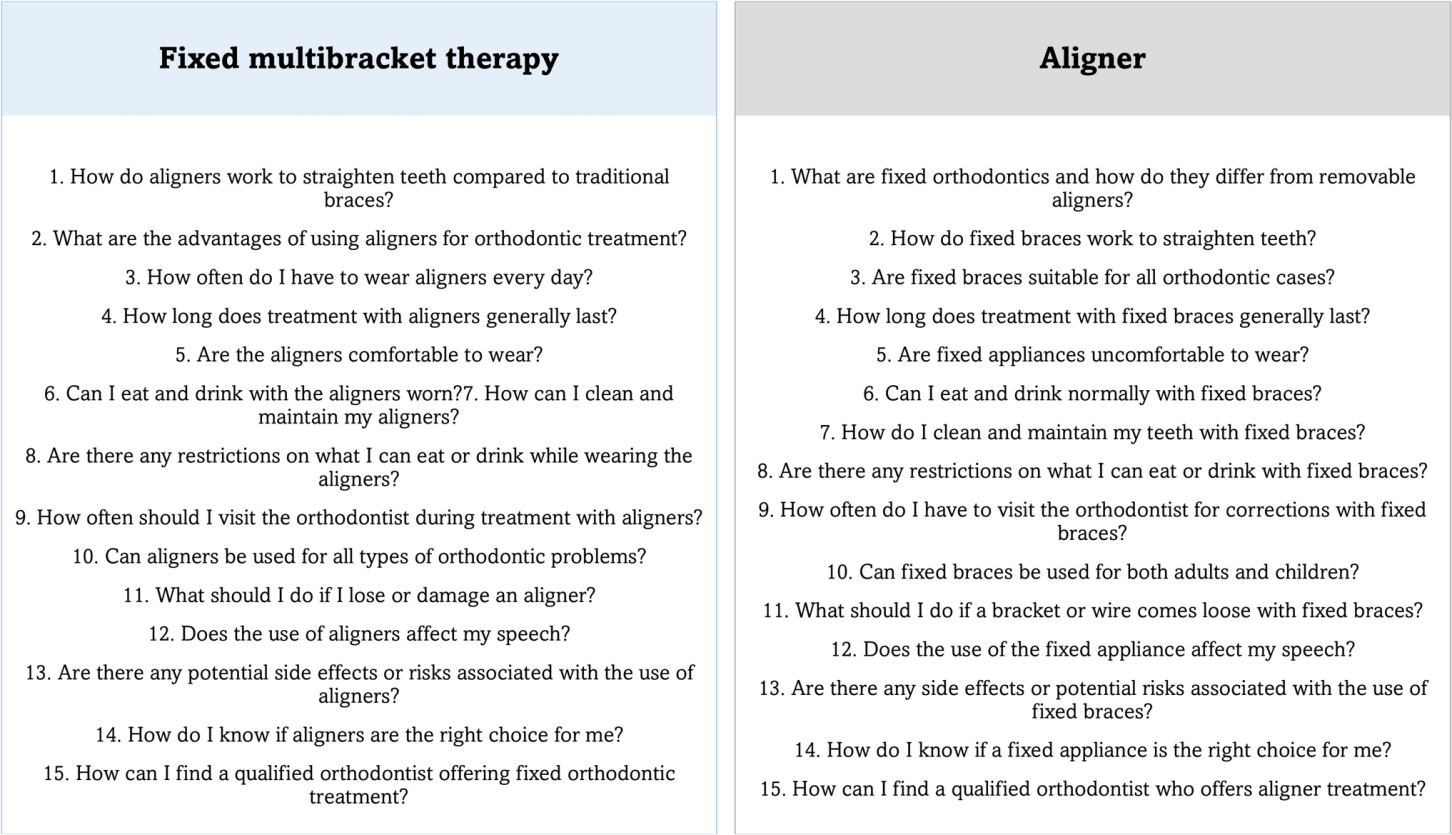
Most frequently asked questions according to the AAO database. The figure was created with Power Point (Microsoft 365 package, Microsoft Corporation, Washington, USA)

The chatbot was created using the free software Botpress (Québec, Canada) (Fig. 4). It was trained by two orthodontic experts to answer common questions about fixed orthodontic therapy and aligners, based on the AAO dataset consulted on May 19 and 27, 2023 (https://aaoinfo.org/treatments/) (Fig. 5). The responses were based on guidelines from leading orthodontic associations *(Sido – Italian Society of Orthodontics, AAO – American Association of Orthodontists*) and official ministerial guidelines. To enhance accuracy and ensure relevance, the chatbot underwent a thorough training process with expert-reviewed content. Feedback loops were integrated into the development phase, allowing for iterative adjustments and refinement based on user interactions and expert feedback. Additionally, the validation process involved cross-checking the chatbot’s responses with trusted orthodontic sources to confirm their consistency with best practices. This iterative training and validation approach aimed to improve the chatbot’s reliability, ensuring that it provides users with accurate, evidence-based responses tailored to their needs.

When a user launches the chatbot, the first interaction begins with a general menu. The menu is made up of 4 options:

1. “I want to know why orthodontic treatment is important”;
2. “I want to have information on a specific orthodontic treatment”;
3. “I want to have information about home oral hygiene procedures”;
4. “I want to ask a question.”

**Fig. 6:**
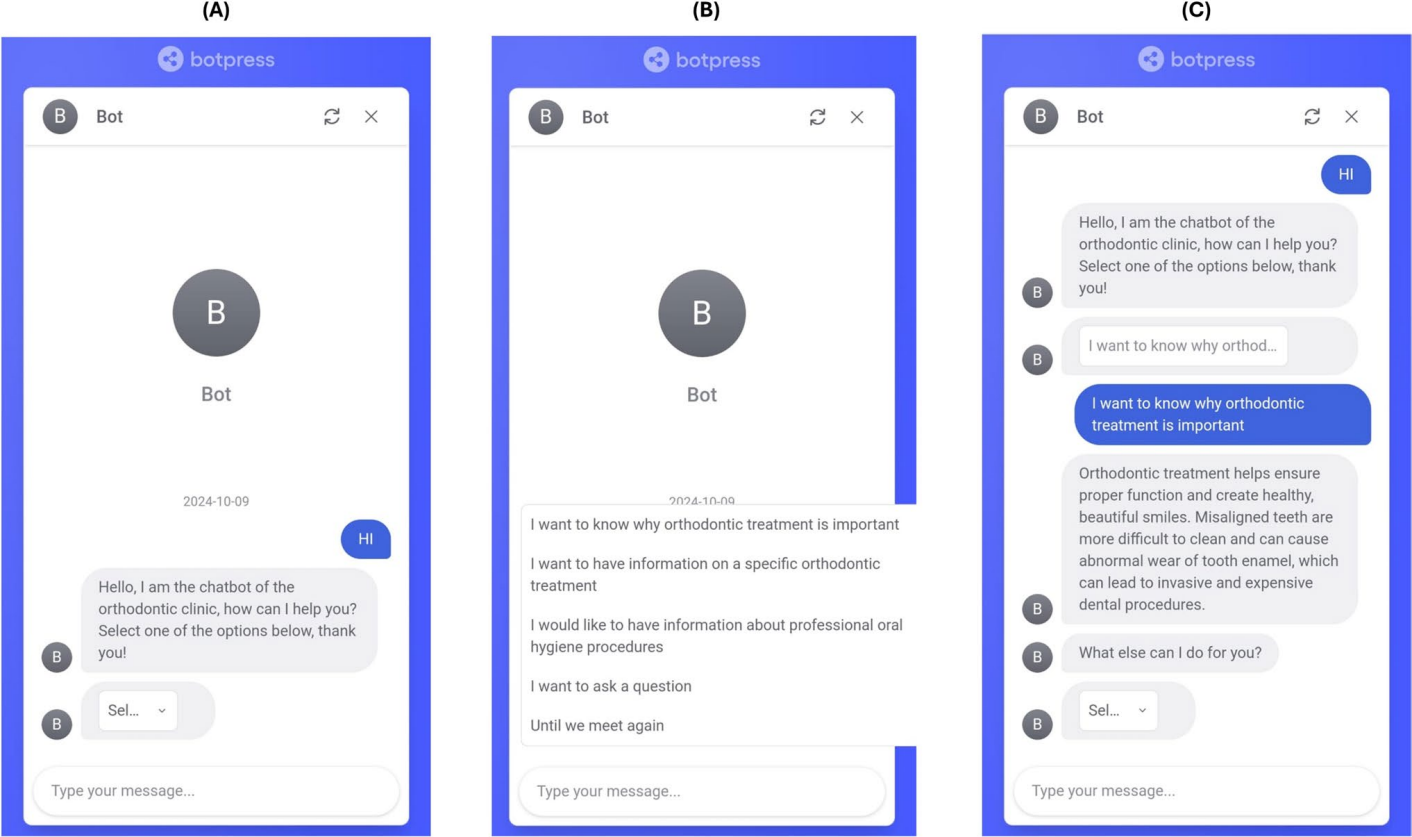
Graphical user interface-chatbot. **A** Main menu execution screen. **B** The user enters the “main menu” on the screen and the chatbot will display the 4 main menu items on the screen. **C** The user can select and execute the option they want, or they can directly enter a request. The figure was created with *Power Point* (Microsoft 365 package, Microsoft Corporation, Washington, USA)

After selecting an option, the user can start the conversation. Once the chatbot has answered, the user can either make further requests or interrupt the session and pick it up later from where it left off. (Fig. 6).

### Power analysis

The power analysis was calculated using an online calculator (EasyMedStat). The number of subjects required was estimated by assuming, based on the existing literature, that the mean of the primary outcome MGI would be 1.45 and 0.9 for the control and intervention groups, respectively, with a common standard deviation of 0.88 [15]. Therefore, with a risk of an α-error of 0.05, a power level of 80%, an allocation ratio of 1:1, a two-sided test, and an estimated drop-out rate of 5%, it was indicated that a minimum of 86 patients (43 per group) would be required.

### Statistical analysis

The results of the scores were filed in Microsoft Software Excel and analysed using a common Statistical Package for Social Sciences (IBM SPSS v.29). Clinical data were expressed as mean and standard deviation (parametric data), median and interquartile range (non-parametric data), or absolute frequencies and percentages (categorial variables). The distribution of quantitative measures was carried out using the Shapiro–Wilk test to determine normality. To quantify the impact between the baseline differences and study outcomes, Chi-square tests are performed for categorical variables (gender, education level, and age ranges) and t-tests for continuous variables (age). For intra- and intergroup comparisons of parametric data, the paired t-test and independent t-test were applied, respectively. In cases of non-parametric data, the Mann–Whitney U test was used for intergroup comparisons. To evaluate the effects of orthodontic therapy on clinical parameters and questionnaire scores between the two groups, post-hoc analyses were conducted using one-way and two-way analysis of variance (ANOVA) for parametric data, and the Kruskal–Wallis test for non-parametric data. The reliability of the questionnaire was evaluated using Cronbach’s α to assess internal consistency. All statistical tests were two-tailed, with significance set at an alpha level of < 0.05, and 95% confidence intervals were calculated for relevant measures.

## Results

At baseline (T0), the sample was unevenly distributed in both groups about gender, age, age range, education levels and score on the KE_S and SS_S sections of the questionnaire. Instead, the distribution of the clinical parameters (MGI, PI), the therapy, the scores of the remaining sections of the questionnaire (US_S and CAAS_S) and the total score of the questionnaire (Q_TOT) are evenly distributed. Tables 1 and 2 show the description of demographic characteristics of sample at baseline (T0) and of respondents’ answers to the questionnaire at T1. There were no differences between treatment groups regarding gender, age, age range, education level and therapy (Table 1).

**Table 1:** Demographic characteristics of the test groups at baseline (T0), expressed as mean and standard deviation or median and max and min (numerical parameters) and as numerical and percentage frequency (categorical parameters)

	Control (*N*° = 20)	Chatbot (*N*° = 20)	*P *Value
Gender, *N*°(%)			0,558**
*Male*	*10 (50)*	*11 (55)*	
*Female*	*10 (50)*	*9 (45)*	
Age, median (max, min)	*34,5 (18, 63)*	*34 (18, 63)*	0,661*
Age Ranges, *N*°(%)			0,529**
* 18–29 years*	*7 (35)*	*8 (40)*	
* 30–39 years*	*5 (25)*	*5 (25)*	
* 40–49 years*	*2 (10)*	*4 (20)*	
* 50–59 years*	*3 (15)*	*2 (10)*	
> 60 years	*3 (15)*	*1 (5)*	
Education Level, *N*°(%)			0,207**
* Secondary school level*	*3 (15)*	*3 (15)*	
* Diploma*	*12 (60)*	*8 (40)*	
* University degree*	*5 (25)*	*9 (45)*	
Therapy, *N*°(%)			0,558**
* Fixed multibracket therapy, (F)*	*11 (55)*	*9 (45)*	
* Aligners, (A)*	*9 (45)*	*11 (55)*	

*T-test for independent variable

** Chi-square test

**Table 2:** Characteristics of respondents’ answers to the questionnaire at T1, expressed as mean and standard deviation or median and max and min (numerical parameters) and as numerical frequency and percentage (categorical parameters)

	Questionnaire									
	Control (*N*° = 20)					Chatbot (*N*° = 20)				
	0	1	2	3	4	0	1	2	3	4
Q_TOT, (M ± DS)	23,90 ± 8,17					28,25 ± 6,69				
	Knowledge Evaluation									
*KE_1, N°(%)*	*5 (25)*	*8 (40)*	*4 (20)*	*2 (10)*	*1 (5)*	*4 (20)*	*8 (40)*	*4 (20)*	*2 (10)*	*2 (10)*
*KE_2, N°(%)*	*3 (15)*	*5 (25)*	*8 (40)*	*4 (20)*	*0*	*2 (10)*	*9 (45)*	*6 (30)*	*3 (15)*	*0*
*KE_S,* Median (min, max)	*3,00 (0, 7)*					*8,00 (1, 11)*				
	Understending Scale									
*KS_1, N°(%)*	*0*	*3 (15)*	*6 (30)*	*10 (50)*	*1 (5)*	*1 (5)*	*7 (35)*	*5 (25)*	*6 (30)*	*1 (5)*
*KS_2, N°(%)*	*1 (5)*	*4 (20)*	*4 (20)*	*7 (35)*	*4 (20)*	*2 (10)*	*5 (25)*	*4 (20)*	*7 (35)*	*2 (10)*
*KS_3, N°(%)*	*1 (5)*	*3 (15)*	*4 (20)*	*9 (45)*	*3 (15)*	*2 (10)*	*4 (20)*	*6 (30)*	*7 (35)*	*1 (5)*
*US_S,* (M ± DS)	*6,05* ± *1,39*					*6,08* ± *3,2*				
	Conformity Assessment and Adherence Scale									
*CAAS_1, N°(%)*	*0*	*6 (30)*	*4 (20)*	*6 (30)*	*4 (20)*	*0*	*0*	*7 (35)*	*8 (40)*	*5 (25)*
*CAAS_2, N°(%)*	*0*	*4 (20)*	*5 (25)*	*7 (35)*	*4 (20)*	*0*	*1 (5)*	*2 (10)*	*10 (50)*	*7 (35)*
*CAAS_S,* (M ± DS)	*4,95* ± *1,90*					*6,05* ± *1,39*				
	Satisfaction Scale									
*SS_1, N°(%)*	*2 (10)*	*3 (15)*	*6 (30)*	*6 (30)*	*3 (15)*	*1 (5)*	*0*	*7 (35)*	*7 (35)*	*5 (25)*
*SS_2, N°(%)*	*0*	*6 (30)*	*6 (30)*	*5 (25)*	*3 (15)*	*0*	*1 (5)*	*2 (10)*	*12 (60)*	*5 (25)*
*SS_3, N°(%)*	*0*	*4 (20)*	*7 (35)*	*2 (10)*	*7 (35)*	*0*	*4 (20)*	*4 (20)*	*4 (20)*	*8 (40)*
*SS_4, N°(%)*	*0*	*1 (5)*	*9 (45)*	*3 (15)*	*7 (35)*	*0*	*1 (5)*	*5 (25)*	*3 (15)*	*11 (55)*
*SS_S,* Median (min, max)	*9 (5, 16)*					*11, 50 (5, 16)*				

### Clinical evaluation

The intra-group comparison of the analysed clinical parameters at T0 and T1 indicated a statistically significant increase in MGI and PI in both the control group (*p* < 0.001) and the chatbot group (*p* < 0.001 and *p* = 0.01, respectively) (Table 3).

**Table 3:** Intra-group comparisons T0 (baseline) and T1 of the clinical parameters analysed

	CONTROL (*N*° = 50)	CHATBOT (*N*° = 50)	
	T1-T0	T1-T0	*P value*
MGI	*1,27* ± *0,08*	*1,2* ± *0,17*	< 0,001*
PI	*0,21* ± *0,11*	*0,15* ± *0,11*	0,185*
Q_TOT	*23,90* ± *8,17*	*28,25* ± *6,69*	0,078*
KE_S	*3,00 (0, 7)*	*8,00 (1, 11)*	0,34**
US_S	*6,05* ± *1,39*	*6,08* ± *3,2*	0,42*
CAAS_S	*4,95* ± *1,90*	*6,05* ± *1,39*	0,12*
SS_S	*9 (5, 16)*	*11, 50 (5, 16)*	0,71**

*T-test for independent variable

**Mann–Whitney U test

The intergroup comparison of clinical parameters indicated that the increase in MGI after initiation of orthodontic treatment was significantly greater in the Control group than in the Chatbot group (*p* < 0.001) (Table 4).

**Table 4:** Inter-group comparisons T0 and T1 of the clinical parameters analysed and the results to the questionnaire

	CONTROL (N° = 50)			CHATBOT (N° = 50)		
	T0	T1	P*	T0	T1	P*
MGI	*0,93* ± *0,12*	*1,27* ± *0,08*	< *0,001*	*0,9* ± *0,01*	*1,03* ± *0,06*	< *0,001*
PI	*0,98* ± *0,07*	*1,2* ± *0,17*	< *0,001*	*0,86* ± *0,08*	*1,02* ± *0,12*	*0,01*

* T-test for dependent variables

To evaluate the influence that orthodontic therapy may have on the two groups analyzed, 4 subgroups were identified: LA, LF, CA and CF. Analysis of variance (uni and multivariate ANOVA) indicated that the variation of GMI was highly statistically significant within the 4 subgroups analysed (LF, LA, CF, CA) (*P* < 0.001), affecting the variation of the data with a power of 74.% (Table 5). Therapy appears to have a large impact (73%) on the variation of this parameter.

**Table 5:** Uni and multivariate ANOVAs between the clinical parameters, the total and section scores of the questionnaire and the subgroups analysed in relation to therapy (LF, LA, CF and CA)

	Intervent and therapy				
	MS	F	R-quadrato	*P*-value	η2
MGI	0,117	18,97	0,74	< 0,001	0,73
PI	0,065	2,620	0,28	0,079	0,29
Q_TOT	9,92	0,113	0,017	0,95	0,02
US_S	2,846	0,298	0,826	0,82	0,83
CAAS_S	0,937	0,285	0,836	0,83	0,84

Tukey’s post hoc survey shows that in the CF and CA subgroups there was a statistically lower increase in MGI (*p* < 0.001) than in the other two subgroups analysed (LF and LA) and that this increase was lower in the CA subgroup than in the CF subgroup (Table 6).

**Table 6:** Post Hoc Comparison—Therapy (LF, LA, CF and CA)

Therapy (I)	Therapy (J)	Difference Of Media (I-J)	Gi	Es	*P* Value*	95% Confidence Interval	
						Min	Max
LA	*LF*	*− 0,2523*	*3*	0,045	*0,943*	− 0,151	0,101
	*CF*	*0,2390*	*3*	0,047	< *0,001*	0,106	0,371
	*CA*	*0,2196*	*3*	0,044	*0,000*	0,097	0,341
LF	*LA*	*0,0252*	*3*	0,045	*0,943*	− 0,101	0,151
	*CF*	*0,2642*	*3*	0,047	< *0,001*	0,131	0,397
	*CA*	*0,2448*	*3*	0,044	< *0,001*	0,122	0,366
CA	*LF*	*− 0,2390*	*3*	0,047	< *0,001*	− 0,371	− 0,106
	*LA*	*− 0,2642*	*3*	0,047	< *0,001*	− 0,397	− 0,131
	*CF*	*− 0,0196*	*3*	0,046	*0,974*	− 0,147	0,109
CF	*LF*	*− 0,2196*	*3*	0,044	< *0,001*	− 0,341	− 0,097
	*LA*	*− 0,2448*	*3*	0,044	< *0,001*	− 0,366	− 0,122
	*CA*	*0,01938*	*3*	0,046	*0,974*	− 0,109	0,147

*Post hoc* of Tukey*

### Questionnaire evaluation

Cronbach’s Alpha, used to calculate the reliability or internal consistency of the questionnaire, indicated that each section of the questionnaire and the questionnaire had a level of reliability within the reference range (0–1) (Table 7). In detail, the Q_TOT shows a strong internal coherence (0.87), while the KS_S section (with two elements) shows a slightly lower value than those of the other sections analyzed, but still acceptable. Overall, the instrument demonstrates good reliability, with most of the sections meeting the ideal threshold of coherence. However, the interpretation of Cronbach’s Alpha must consider that although values ​​between 0.7 and 0.9 indicate good reliability of the questionnaire, values ​​above 0.9 could also be indicative of redundancy and similarity between the items constituting the questionnaire. Furthermore, Cronbach’s Alpha tends to increase with the number of items on a scale, so a questionnaire with few items could produce lower Alpha values ​​even if those items are conceptually strong.

**Table 7:** Alpha cronbach test

	Alpha Cronbach
Q_TOT	0,87 (*n*° elements = 11)
KS_S	0,75 (*n*° elements = 2)
US_S	0,82 (*n*° elements = 3)
CAAS_S	0,88 (*n*° elements = 2)
SS_S	0,84 (*n*° elements = 4)

No statistically significant differences were observed when the sums of the questionnaire section scores and the total score of the evaluation questionnaire were compared (Table 4). The different therapies had no statistically significant effect on the partial scores of the questionnaire sections and the total score of the questionnaire (Tables 5 and 8).

**Table 8:** Kruskal–Wallis test between subgroups analysed in relation to therapy (LF, LA, CF and CA) and the S_SS session of the questionnaire

	Intervent and therapy			
	Median (Max, min)	gl	${X}^{2}$	*P*-value
*KE_S*	*3 (0,7)*	*3*	*1,001*	*0, 56*
*SS_S*	*11 (5,16)*	*3*	*1,034*	*0,79*

## Discussion

This study compared the level of oral hygiene and knowledge and understanding of the therapeutic recommendations received and the degree patients’ satisfaction of orthodontic patients undergoing fixed multibrackets or aligner therapy, who received information regarding treatment and oral hygiene procedures either via information leaflets or via an AI-based chatbot created by experts in the field. At T0, both groups had MGI and PI values lower than 1 (Table 1) and, therefore, compatible with gingival health and a good level of oral hygiene. At T1, both groups showed a statistically significant increase in the clinical parameters MGI and PI (*P* < 0.001 and *P* = 0.01), in agreement with previous studies indicating a worsening of oral hygiene levels immediately after the start of orthodontic treatment [1, 6]. However, in the chatbot group, the increase in MGI was statistically lower than that observed in the brochure group (*P* < 0.001). PI also showed a lower increase in the chatbot group than in the control group, which, however, was not statistically significant. One possible explanation for the findings is that patient-chatbot interactions, mimicking a dialogue between individuals, are an enabler of knowledge generation due to increased learner engagement and instant feedback and support [19]. According to a recent systematic review, in fact, AI-based chatbots have proven effective in inducing health behaviour change among large and diverse populations, offering patients a non-judgmental space for communicating sensitive information [20, 21]. A systematic review exploring the role of e-health and AI in improving orthodontic care also indicated that digital platforms, including AI-based chatbots, can improve patient education, treatment adherence, and overall satisfaction by providing instant and accessible support to patients during treatment [22]. Therefore, based on what has been said and the context analysed, the chatbot would represent a virtual assistant to the orthodontist, capable of providing constant educational support during orthodontic treatment, instantly providing answers to patients’ queries, anywhere and anytime, without the need to contact the specialist, with an improvement in patient compliance [21, 23]. Recent studies carried out in Japan, which used an AI-based chatbot to help chronic low back pain sufferers, indicated that its use increases patient adherence to specialists’ instructions with a consequent reduction in pain [24, 25].

The subgroup analysis, obtained considering the type of orthodontic treatment (LF, LA, CF and CA), indicated that orthodontic therapy has a statistically significant effect on the change in the MGI parameter (*p* < 0.001) in the two treated groups. In detail, it was observed that in the CF and CA subgroups there is a statistically significant greater reduction in MGI than in the LF and LA subgroups (*p* < 0.001) and that this reduction is greater in the CA group than in the CF group (Table 7). These results are in line with previous studies in which it was observed that PI, PD, BOP, FMPS and FMBS scores were significantly lower and compliance with oral hygiene was significantly higher in the aligner group than in the fixed multi-bracket therapy group [11]. It is known, in fact, that the presence of brackets on the surface of the tooth makes cleaning the teeth more difficult, increases due to the intrinsic difficulties linked to accessing the surface of the tooth with normal hygienic instruments, and involves an increase in time and energy needed to debride adequately all surfaces of the teeth [26].

Although previous research has shown that digital health tools, such as mobile apps and online educational resources, significantly increase patient adherence to treatment protocols and increase overall patient satisfaction by providing convenient, real-time access to information and support [27], the analysis of the answers to the questionnaire did not make it possible to determine whether the increase in scores, indicating greater patients’ knowledge, adherence, and satisfaction, in the chatbot group compared to the control group was the result of the interventions analysed or chance. However, these results must be interpreted with caution. Indeed, although Cronbach’s alpha indicated a good reliability of the questionnaire administered, Likert scales can be subject to several types of bias [18], as they lend themselves to possible ‘manipulation’ by respondents (although the polarity of the answers was modified for each question in order to reduce possible automatisms in the participants’ answers) and are closely related to how the questionnaire was designed and devised. Patient motivation is a significant factor that can impact adherence to treatment protocols, potentially influencing the observed outcomes. Highly motivated patients, regardless of whether they received information via a chatbot or a leaflet, may report better outcomes due to their intrinsic drive to improve their oral health. External factors, such as family support, peer influence, and access to other professional care, may also contribute to the results, making it difficult to isolate the impact of the chatbot alone.

The limitations of the study included the following:

- Short observation period: The study only provides an initial view of the effectiveness of the interventions and does not allow for an assessment of how patient adherence to recommendations might evolve over a longer period.
- Use of a Likert questionnaire: This method was employed to assess patient knowledge, compliance, and satisfaction. However, Likert scales may introduce response biases, which could affect the accuracy of the results. Using patient interviews as an additional evaluation metric could overcome the limitation in the use of Likert scales.
- Lack of consideration for malocclusion types: The study did not account for the type of malocclusion, which could influence how well patients follow instructions and how effective the chatbot is in providing appropriate guidance.
- Failure to monitor procedural issues: The study did not track potential complications that may arise during the observation period, such as brackets detaching, changes in the arch, or the loss of aligners. These issues could significantly affect the outcomes and patient compliance.
- Rigid chatbot response programming: The chatbot’s responses are predetermined and categorized into four fixed options from the outset. This rigid structure could limit the chatbot’s ability to provide accurate, contextually relevant, and tailored information, particularly in complex orthodontic cases where customization is critical. Such limitations may influence both the trustworthiness and effectiveness of the chatbot in supporting patient care.

## Conclusion

In conclusion, from the results of the present study it can be deduced that the use of an AI-based chatbot, whose reliability of the information provided can be validated, positively influences patient compliance in terms of adherence to oral hygiene manoeuvres. Although the questionnaire scores were higher in the chatbot group, it could not be established whether the AI-based chatbot positively influences the levels of knowledge and understanding of therapeutic information and the patient’s level of satisfaction with the information received. compared to patients who received information leaflets. Therefore, further studies with longer follow-ups taking into account the types of malocclusion and external factors influencing patient motivation are needed to better understand the impact chatbots may have on the management of orthodontic patients.

## Data Availability

No datasets were generated or analysed during the current study.
